# End User–Informed Mobile Health Intervention Development for Adolescent Cannabis Use Disorder: Qualitative Study

**DOI:** 10.2196/13691

**Published:** 2019-10-04

**Authors:** Kara Bagot, Elizabeth Hodgdon, Natasha Sidhu, Kevin Patrick, Mikaela Kelly, Yang Lu, Eraka Bath

**Affiliations:** 1 Department of Psychiatry University of California, San Diego La Jolla, CA United States; 2 Department of Family and Preventive Medicine University of California, San Diego La Jolla, CA United States; 3 Neuropsychiatric Institute University of California, Los Angeles Los Angeles, CA United States; 4 University of California, San Diego La Jolla, CA United States

**Keywords:** adolescent, cannabis, mobile health, treatment, smartphone

## Abstract

**Background:**

The rates of cannabis use continue to increase among adolescents and the current interventions have modest effects and high rates of relapse following treatment. There is increasing evidence for the efficacy of mobile technology–based interventions for adults with substance use disorders, but there is limited study of this technology in adolescents who use cannabis.

**Objective:**

The goal of our study was to elucidate elements of an app-based adjunctive intervention for cannabis cessation that resonate with adolescents who use cannabis.

**Methods:**

Adolescents, aged between 14 and 17 years, who used cannabis were recruited from San Diego County high schools. Semistructured focus groups (6 total; N=37) were conducted to examine the ways in which participants used smartphones, including the use of any health behavior change apps, as well as to elicit opinions about elements that would promote engagement with an app-based intervention for adolescent cannabis cessation. An iterative coding structure was used with first cycle structural coding, followed by pattern coding.

**Results:**

Themes that emerged from the analysis included (1) youth valued rewards to incentivize the progressive reduction of cannabis use, which included both nontangible rewards that mimic those obtained on social media platforms and prosocial activity-related rewards, (2) having the ability to self-monitor progression, (3) peer social support, (4) privacy and confidentiality discrete logo and name and usernames within the app, and (5) individualizing frequency and content of notifications and reminders.

**Conclusions:**

Integrating content, language, interfaces, delivery systems, and rewards with which adolescents who use cannabis are familiar, engage with on a day-to-day basis, and identify as relevant, may increase treatment engagement and retention for adolescents in substance use treatment. We may increase treatment effectiveness by adapting and individualizing current evidence-based interventions, so that they target the needs of adolescents and are more easily incorporated into their everyday routines.

## Introduction

Cannabis is the most prevalent drug of abuse among adolescents, with nearly 50% of 12th graders, 30% of 10th graders, and 15% of 8th graders reporting lifetime use [1]. Although the rates of use of many substances have been on the decline among youth, cannabis use has failed to decline [1]. Greater than 25% of adolescents who use cannabis meet criteria for cannabis use disorder (CUD) [2]. The likelihood of developing cannabis dependence is linearly associated with the frequency of use and inversely associated with age, such that early onset users are the most susceptible for later dependence [3]. This is of concern as adolescents’ brains are developing and more susceptible to perturbations because of substance exposure. Early exposure to high levels of delta-9-tetrahydrocannabinol through cannabis use triggers repeated activation of the endogenous mesolimbic dopaminergic system, which may in turn lead to sensitization of this system and progressive enhancement of acquired susceptibility to psychiatric illness [4]. Early regular cannabis use is associated with psychosocial consequences that increase burden of illness and decrease functional outcomes [5,6], including increased likelihood of other illicit drug use and poorer academic outcomes [7].

To date, the evidence for efficacious interventions targeting problematic cannabis use, especially in youth, are sparse, with some behavioral interventions demonstrating short-term abstinence during active treatment, but high rates of relapse at follow-up. Specifically, randomized controlled trials of behavioral interventions for adolescent cannabis use show moderate effect sizes posttreatment for motivational enhancement therapy (MET), cognitive behavioral therapy (CBT), family support therapy, case management, and contingency management (CM), with few adolescents maintaining abstinence through follow-up [6,8]. In a study evaluating CM plus MET and/or CBT on cannabis use outcomes in adolescents, those youth assigned to active treatment demonstrated longer durations of continuous abstinence during treatment and higher rates of point prevalence posttreatment abstinence as compared with behavioral treatment alone [9]. Further study of adjunctive CM has yielded conflicting results [10]. In addition, 5-week and 12-week MET and/or CBT, family support with home visits, psychoeducation, case management and referral to self-help groups, adolescent community reinforcement approach (operational skills training+social systems support), and multidimensional skills therapy have all demonstrated initial within-intervention efficacy, with reduction of use and increased abstinence. However, posttreatment, adolescents experienced frequent cycles of recovery and relapse; many were unable to maintain abstinence and the majority continued to report substance-related problems at follow-up [11]. This suggests that continued monitoring and intervention following active treatment may be important in maintaining gains during treatment.

Studies suggest that the effectiveness of interventions may be increased by developing selective, highly specific interventions that are presented on interactive platforms and include content addressing specific risk factors in at-risk youth [12]. However, few interventions have done so to date [13]. Mobile phones are a highly promising and palatable intervention platform for adolescents as access to mobile technology is ubiquitous; nearly 75% of adolescents own or have regular access to a smartphone (85% African American and 71% Hispanic and Caucasian) and 91% access the internet through a smartphone. Furthermore, 90% of adolescents with mobile phones use text messaging, sending and receiving an average of 30 messages daily [14]. Minority adolescents appear more likely to own smartphones and use apps, and those who are most likely to own a smartphone are also those in need of behavioral change interventions [15]. Therefore, there is enormous potential for leveraging mobile technologies to widely disseminate interventions for adolescent substance use. Furthermore, the ubiquity of mobile technology use among African American and Hispanic teenagers, provides the opportunity for increasing substance use treatment and public health intervention among historically underserved populations.

Although, adolescents spend a significant amount of time using technology, are expert users by virtue of exposure and use from childhood, and interactive platforms have been shown to be effective for treatment delivery in youth [12,14], few mobile health (mHealth) interventions (internet/mobile phone–based platforms) have been developed for use in adolescent substance users and none for adolescent cannabis use [16]. As such, we look to the adult data for guidance in examining the potential applicability of these types of interventions for at-risk adolescents. *CBT4CBT* (a computer-based CBT program supplementing therapist-delivered CBT has been shown to have greater efficacy (more days abstinent and fewer positive drug screens) than therapist-delivered CBT alone, for adults with SUDs up to 6 months posttreatment [17,18]. Web-based self-help interventions have also been shown to be efficacious in reducing alcohol use in adults [19-21]. Limited study of Web- and computer-based interventions in cannabis-using young adults and adults have demonstrated efficacy in correcting misperceptions about use and increasing knowledge but demonstrate mixed results for cessation outcomes [22-25]. Adults in a study of the *Therapeutic Education System* (self-guided, Web-based CBT modules+CM for completing modules and abstinence), exhibited higher rates of abstinence and greater treatment retention at study completion as compared with treatment-as-usual (TAU) [26]. A Web-based, self-guided intervention called *Reduce Your Use: How to Break the Cannabis* *Habit*, which comprises modules based on CBT, MI, and behavioral self-management, demonstrated significantly greater rates of retention and reduction of cannabis use, frequency and quantity, during intervention as compared with controls [27]. The *Quit the Shit* intervention (52 weeks; Web-based questionnaires, weekly cannabis use diary with feedback, baseline and posttreatment therapist *chat*) also demonstrated a reduction in frequency and quantity of cannabis use in adults [28].

The goal of this study is to increase our understanding of adolescent cannabis use behaviors in the context of near universal availability of mobile, wireless, and wearable technology, with the overall aim of developing a user-informed mHealth intervention for high-risk cannabis-using adolescents engaged in outpatient substance use treatment. Specifically, we aimed to (1) examine adolescent use of various mobile platforms (ie, short message service [SMS] text, apps), social media, and wearable devices to determine the use of mobile technologies as sources of health and substance use related information, and the effect on risk-taking behaviors and behavioral change, and (2) examine salience of components of existing behavioral interventions for youth.

## Methods

### Recruitment

We employed a stratified purposeful sampling strategy. A total of 37 adolescents aged between 14 and 18 years of age were recruited from high schools and substance use treatment centers in San Diego County, and San Diego Unified School District (SDUSD), which serves >130,000 students and is the second largest district in California. The racial/ethnic makeup of the students in the SDUSD is 46.5% Hispanic, 23.4% Caucasian, and 10.2% African American. Eligibility criteria for the focus groups included being 14 to 18 years of age, owning a smartphone and/or having internet access, speaking fluent English, and cannabis use one or more times in the past 30 days. If eligible, adolescents were invited to a focus group held either on their school campus, in the outpatient treatment center, or in the lab.

A total of 37 adolescents were enrolled in one of 6 focus groups, as that was the number needed to reach saturation (themes were repeated, no new information obtained) [29]. Groups were stratified on the basis of age (early adolescence: 14-16 years/8th-10th grades; late adolescence: 17-18 years/11th-12th grades) so that younger adolescents would not feel intimidated to speak in the presence of older teens. Age stratification was also done on the basis of data that marijuana use differs significantly by adolescent age, and the belief that differing rates of use and exposure to methods and strains of marijuana use might influence their experiences and ideas about a cannabis intervention. The groups included a maximum of 10 and a minimum of 5 adolescents per group so that the groups were small enough to give each participant the opportunity to speak and share ideas but large enough for some diversity. Adolescents were compensated with a US $25 gift card and travel to/from the focus group site was provided, if needed. Institutional Review Board approval and a Certificate of Confidentiality from the National Institutes of Health were obtained.

### Design

Focus groups were conducted in English and followed a semistructured interview guide developed by the study principal investigator, a child and adolescent psychiatrist. Participants answered questions regarding characteristics of their smartphones, how they use their phones, what they use them for (including apps downloaded and native apps), time spent on their phones, use of related mobile technologies such as activity trackers, and use of mobile technology and Web-based platforms for accessing, monitoring, and/or changing health or substance related behaviors. Participants also worked as a group to design, and arrive at a consensus among group members, a mock app-based cannabis intervention for cannabis-using adolescents. They were prompted to discuss aspects of interest, including the following:

1. Content
  - Health-related content for in-app messages and/or push notifications
  - Access to psychoeducation and prior measured personal data (eg, cannabis use/treatment adherence)
  - Static (same intervention for all users) versus dynamic (individualized/tailored) messaging
  - Theory: learning (temporal feedback on individual level data to enhance engagement and behavior change) and social cognitive (increased self-efficacy with goal setting and defined expectations, positively reinforce behavior change)
2. Usage
  - Frequency of content delivery (exposure) via prompts, cue-related, global positioning system (GPS) location triggered
  - Format of content: text, video, pictures, and/or links to external sources
3. Duration: active (brief, extended) with or without continuing care (time limited vs indefinite access)
4. Contact
  - Clinicians via SMS text and Skype/video for confirmation of abstinence at routine intervals and in response to psychiatric or high-risk behavioral concerns
  - Peer supports via in-app social media
5. Accessibility
  - Navigational features
  - Design of user interface
  - Environments (feasibility of continuous monitoring/access to intervention across academic, social, and familial settings).

### Measures

Participants were administered 4 self-report questionnaires: the Customary Drinking and Drug Use Record [30] to measure current and past substance use, an internet and a social media addiction scale (based on the Bergen Facebook Addiction Scale) [31], a smartphone attachment index (scale from 1 to 10 of the query *How attached are you to your smartphone?* with 10 being the highest), and a social media engagement questionnaire (queries related to time spent and actions taken on various platforms, including Instagram, Snapchat, YouTube, and Facebook). Table 1 presents participant characteristics, mean cannabis use, and phone attachment. Group discussions provided qualitative reports of how adolescents use mobile technology to communicate and track health behaviors, in addition to app development input. Audio recordings were transcribed verbatim and coded individually.

**Table 1:** Participant characteristics.

Demographic information			Values
**Gender (n=37), n**			
	Female	22	
	Male	15	
Age (years), mean (SD)			16.86 (0.82)
**Race (n=37), n**			
	White/Caucasian	32	
	Black/African American	2	
	Not reported	3	
**Ethnicity (n=37), n**			
	Hispanic/Latino	29	
	Not reported	1	
**Smartphone and cannabis use, mean (SD)**			
	Phone attachment index (1-10)	6.97 (2.00)	
	Number of days cannabis used in past 30 days (n=24)	3.04 (5.20)	

### Statistical Analysis

Qualitative software, ATLAS.ti (Scientific Software Development GmbH) was used to aid in data management for thematic analyses of transcripts and coding [32]. We used a team coding approach with iterative coding. Coding was framed by the focus group guide developed following individual interviews of high-risk cannabis-using teens that helped to triangulate information obtained from the focus groups. Themes were selected a priori to correspond to the guide. These themes included health behavior change technology and components of platform design for mobile technology intervention development. A total oftwoindependent coders (KB and EH) determined the coding structures, coded the 6 focus group transcripts, and engaged in intensive discussion of the coded transcripts to refine the coding guide and arrive at consensus in instances of discrepancy. Code cooccurrence tables were analyzed for connections between codes and participant statements. We ensured rigor by checking coding through qualitative software (ATLAS.ti); staff were trained in qualitative methods and coders arrived at a consensus greater than 90% of the time.

## Results

### Health Behavior Change Mobile App Use

Group participants spoke about their use of various smartphone apps, including mHealth apps aimed at changing health behaviors such as sleep or nutrition (Table 2). Participants indicated they had used at least one mHealth app since owning a smartphone. Health behavior change app use was discontinued because of loss of interest or motivation, or dissatisfaction with the features. Wearable activity tracking devices were less popular, viewed as *uncool* and *obsessive* by some, and only useful when a person was dedicated to physical activity (ie, belonging to a sports team).

**Table 2:** Smartphone use: health behavior change technology.

Health behavior change devices and apps			Quotes
Use of wearable devices			“I have the Apple watch that I use for running too. And the Fitbit.”; “I have an Apple watch but I hardly ever go running.”; “[I use] a watch...to track my steps.”
Use of activity tracking apps			“I used to use the health app that [iPhone] comes with...See how much I’ve walked and stuff.”; “It’s called Lifesum...it tracks your calories for the day...I’ve seen some other people using and I was like, ‘that’s pretty cool.’”
**Reason to use health apps or devices**			
	Positive feedback	“All of those big numbers.”	
	Physical training	“Like [if] I’m going to train...”; “[Using a wearable device] depends if I’m doing a sport.”	
	Interface	“I just liked the way it looked, the layout...when you go on there, they ask you a whole bunch of questions. And then they give you a certain amount of calories for your weight and your height and like your age. And then you put in what you eat throughout the day...And then it’s like a rainbow at the top. And it’s like a sunset at the top and then water—I don't know. it’s just a cool setup, I guess.”	
**Reasons not to use health apps or devices**			
	Pressure to workout	“Because it’s too much exercise...because I would want to get big numbers.”	
	Uncool	“It’s sort of just inherently uncool to me.”	
	Fear of losing device	“I feel like I’d lose it.”	
**Discontinuation**			
	Inconvenient/no new insight	“[Sleep app] I don’t like to have my phone by my bed because it lights up and stuff. It had to be like pretty close to you for it to track...I mean like it was [useful]. It told me if I was sleeping good or not. But I’m also a really good sleeper so it always was like deep sleep and I’m like okay.”	
	Not necessary	“I just didn’t feel the need to have it. It was something to do.”	
	Lost interest	“Well, at first, I would read them because it would just pop up, but then after like I would read them and they would just keep popping up and I wouldn’t care and I would just swipe up. So, I was like oh I didn't even read them. So, I deleted it.”	
Perceptions of those who use cannabis			“Fitbit is like a preppy thing. It’s the kids that would be willingly spend $100 for something a phone app can do.”; “I’m just like oh my God, what a nerd. I just do not want to be associated with someone who is like, ‘Oh, I’ve got to get my steps in today.’”; “It’s a little obsessive to me.”; “At least they’re motivated.”

### Mobile App Design Features

During the group discussion, the following topics were considered: (1) content, (2) usage, (3) duration, (4) contact, and (5) accessibility. Themes from each area were derived through coding of focus group transcripts and are summarized in the following tables. The most prominent themes among participants were rewards, privacy, self-monitoring, peer social support, and notifications.

#### Rewards

Participants discussed creating a reward system for cannabis use cessation (Table 3). Participants proposed a contingency system of incremental rewards for sustained abstinence. Monetary rewards, such as prepaid debit cards, gift cards, coupons, or discounts, were suggested by participants across groups. In addition, participants proposed in-app rewards, such as points, emojis, pictures, exclusive app features, and games, as an alternative to monetary prizes.

**Table 3:** Preferences and design recommendations for an mHealth app for cannabis cessation: reward system.

Rewards			Quotes
**Types of reward**			
	In-app rewards	“I don’t think it has to be anything special. You know how like on Snapchat you get the random trophies...No one really cares about them but it’s kind of like I check it. And I’m like oh what did I get? What have I done?”; “...funny pictures.”; “You get cooler stickers.”; “It’s like every time you say you ‘no’ [to using marijuana, you] get a coin...like a game...that you can customize.”	
	Gift card/prepaid debit card	“Yeah, a gift card to Starbucks. Something that you can district you.”; “You can’t go to a [drug dealer] with a credit card.”; “[Gift cards for] Wal-Mart. Target. Regular shit.”	
	Cash	“Money...Ten bucks or fifteen. Twenty.”	
	Alternative activities	“I feel like with the app you guys should put in like events are happening around us. So it takes our mind off. You know, if we want to go somewhere else and do something it will take our mind off of wanting to smoke.”	
	Coupons	“Maybe even tying in stuff from like Groupon where it gives you discounts to places.”; “I like how they did like coupons or I don't know just something small.”; “Or a coupon for like iTunes or something.”; “...you can achieve a discount.”	
	*Streak*	“You should incorporate like the streaks to your app...Streaks make everyone want to have to do, and on Snapchat they have a little hourglass if you’re about to lose your streak. And once you see the hourglass, you’re like, oh you better snap them. It becomes really important.”; “[No marijuana use] then you just get a sticker or something or a streak to like whatever.”; “Streaks make everyone so much more into it.”	
**Reward frequency**			
	Contingent reward for staying *clean*	“Depending on how many days you stay clean you get certain rewards. And if you report that you did smoke then it will deduct points or deduct rewards.”; “Send you a notification like hey, you’ve been clean for a week. This is what you get, this amount of points. And two weeks you can get like double the points.”; “It probably goes up every month...Every month you stay clean.”; “...if you say two weeks in the app that you’re clean and you haven’t smoked like you could get one...You should get rewarded.”	
	Frequency needed to stay motivated	“Once a week.”; “Every day.”; “I feel like a month is good.”; “Yeah, you guys could do one every week, a different one every week and you get a little bit every day.”	

#### Privacy

Several issues were raised about the protection of users’ private information. Privacy considerations are presented in Table 4. Participants agreed that the app name and logo should be discrete to minimize the chance of parents or friends learning about their cannabis use. Password or passcode protection was another common recommendation.

Attitudes toward the use of location tracking in the app were divided. Supporters of location-based features liked the prospect of regional activity recommendations and a localized social support system. Dissenters argued that privacy was more important than local connection and they felt uncomfortable being monitored. A consensus was reached that the app should allow users to control location permissions.

**Table 4:** Preferences and design recommendations for an mHealth app for cannabis cessation: privacy features.

Privacy	Quotes
Connection to other social media accounts	“I definitely think it shouldn’t post [to] Facebook, ‘I haven’t smoked in two months!’”; “And a lot of the times, when you join an app they’re like find your friends on here. And they download your friends from your contacts. I don't think you should do that.”; “You definitely shzould be able to connect through Twitter, Facebook.”
Concern about global positioning system (GPS) tracking	“It’s just weird to know that somebody could be tracking you wherever you are.”; “Yeah, you could be at like at a club and be like where the fuck are you at?...You work for the FBI or what?”; “I wouldn’t want the GPS for privacy reasons, you know. Some people are weird.”
Anonymous user account	“Pick a user name...So, you wouldn’t have to use your actual name.”; “I think a user name would be the best. Just somehow it doesn’t track back to actually you, never knowing actually who it is.”; “Yeah, it has to totally be private because if a person sees you are on there and she’s like why is she on there, oh, they’ll tell people about what you’re doing.”; “I think user names, but there should be like one where if you want a person to know who you are, [you could].”
Passcode/fingerprint access	“Like when you open your phone, you know, like how there’s a password, you can have a password for the app.”; “You can use your fingerprint to open it.”; “That would be kind of gnarly to put your fingerprint for something you want to stop smoking weed...More like a password. Something you remember like a four digit.”
Discreet app name and logo	“You’d want it to be kind of discreet because a lot of kids have their parents check their apps they download.”; “The logo on the app shouldn’t be a marijuana leaf...it should be a discreet logo if your parents look at your phone, they don’t just see, like—and the name shouldn’t be something like, ‘I’m quitting marijuana.’”

#### Self-Monitoring Cannabis Use

Participants suggested incorporating a feature for users to track their cannabis use. Various tools for collecting user information were proposed, including pop-up prompts and open-ended journal entries (Table 5). Personalized feedback in the form of text messages or graphic representations of cannabis use over time were presented as a way for users to view their progress.

A few group members voiced a need for abstinence verification. Solutions included in-person urine screening, remote drug tests, or providing an electronic testing device that directly attaches to the user’s smartphone.

**Table 5:** Preferences and design recommendations for an mHealth app for cannabis cessation: self-monitoring cannabis use.

Self-monitoring	Quotes
Tracking marijuana use	“I think but the main goal is just to have progress, it’s just to know like you’re staying clean. So, I feel like it’s recording if you’ve done it and...like whenever you’re pressured and you said no, because those are skills to build.”; “I think if you did smoke one day it should ask you questions like how you felt differently than when you did.”; “Or even how often you wanted to smoke but didn’t...When it used to be like oh I wanted to smoke three times, but now I only wanted to smoke once. Like either way it’s progress.”
Journaling	“...you need to know how to...when to say no, and when is the right time to do something.”; “You have like a notepad, where you can write like this thing happened on this certain day and that’s why I did it...and what I could learn from that and what could I change for the next time something that could happen.”
Personalized feedback	“When you put the questions like how do you feel when you smoke? And they give you a response you could be like...or you could feel that way if you do this? Like I feel relaxed, like stressed out. And then you can be like ‘get a soccer ball and you’ll feel relaxed.’”; “Yeah, maybe put it in there so they could see like your ratio of in a month or like a year like how many times did you smoke? And if it’s affecting your health or anything.”
Verifying abstinence	“Send in some stuff. You’ve got to send in some, I don’t know, some pee.”; “Or like at the end have a meeting with them...”; “Why not that little detector like the alcohol detector? You get the app and then they send it to you.”
Providing outside professional resources	“Maybe the app could provide professional help...Like really, really good resources like therapists that they offer or something or that they know of around where they’re at.”; “You know how some apps have the help, you know, like if they really want help. You put that under like oh here’s some places that you can go to get help or something like that...A hotline.”; “If they don’t want to stop or something, that’s when the hotline could come in. And then you like offer that talk. And then you talk to the person. And then if you see no hope then you tell them to talk to somebody else. That’s when you offer the best help.”

#### Peer Social Support

Every group proposed a social network type of messaging to anonymously connect with other app users who were actively trying to stop cannabis use. There was overall approval for the inclusion of social contact with other app users, with precautions taken to protect anonymity and location disclosure. Participants felt that including a social component served as a means of peer encouragement and distraction when cravings arise. Social support features are described in Table 6.

#### Notifications

Participants expressed a desire to have control over the frequency and content of the app notifications (Table 7). The upper limit mentioned by group participants was 3 times a day, though most preferred no more than once a day. When speaking about other mobile app use, participants expressed annoyance with receiving too frequent notifications and subsequently ignoring or turning off all notifications from the app, or deleting the app.

**Table 6:** Preferences and design recommendations for an mHealth app for cannabis cessation: peer social support.

Peer social support	Quotes
Talking to other app users	“Being able to talk to someone else maybe. But create a user name and you don’t have to use your real name. That way if there’s two people who are trying to quit, they can get together and like text each other.”; “Part of the reason a lot of people don’t stop is because they have a certain group of friends that are doing it. So, if they met someone else who was trying to stop, they would be like oh let’s go do this instead of smoking with other friends. Create new friendships...Learn how to do other stuff with new people.”; “There should be a forum for everyone to be able to join in on. And then separately it’s like users, you can just click on them. And then if they have the option of being able to talk to other people because they want then okay, they should be able to.”; “I think it would be really good to talk anonymously to teenagers, like, about trying to quit...Or you can put you in like a random chat...”; “And maybe you can join, like, a group? Like, a group so if you don’t want to be with the others...”
Location-based chats	“I think it would actually be pretty cool if we have like location-wise, like, people around you, like in your city, it would even be pretty interesting to add to that.”; “Let’s just say San Diego has its own group chat and then there’s, like, L.A. and there’s, like, basically the big, major cities.”; “There should be a thing like these people are in your location type of thing like when you have your location on...like these people who are also using the app are around you.”

**Table 7:** Preferences and design recommendations for an mHealth app for cannabis cessation: notifications.

Notifications			Quotes
**Notification delivery**			
	Frequency	“[...once a day would be the most frequent?] Or two times a day maybe.”; “Maybe three. When you wake up, before you go to sleep and the middle of the day.”; “I don’t think it should be like constant reminder of like you’ve been clean for this amount because that will keep you thinking of like still weed. So, it should have it probably once every couple of weeks. And then probably have other motivational things.”	
	Timing	“Like when you’re out partying or something like that. You know, you’re just in the moment of feeling good. Sometimes people smoke when they’re in the vibe too...Or maybe get more notifications at that point on.”	
	User control	“There should be a setting where you can like say you can press I want to check my own notifications. Or I want notifications every two weeks or like every day. Something like that.”; “[You want control over the content of the notifications…] And how often you get them.”	
**Notification content**			
	Reminders of progress	“It would be kind of cool if every three or so days or if it got like a long time like every five like how many days you’re clean because it’s kind of like a reminder. Like okay I’m clean I can’t mess this up, I need to keep on this...it’s three days. Let’s get to five.”; “There should be two different types of reminders and one of them is just to motivate you not to. And then the other one is just check in for tonight and it reminds you at night.”	
	User control	“I would want independent control...cause I would know I could change it if I wanted to.”; “They should also have the option of what kind of information the notification they're giving out. So, for example, some people probably don’t...wouldn’t want for the app to be telling them how bad it is to smoke or something. But some others would find it more motivational too, for the app to tell you oh, this is bad for you because of this or whatever.”; “Maybe for inspirational...Maybe it could be designed into how you want it be. Because reminder quotes people will find irrelevant. So, when you’re getting an inspirational quote you can kind of design it to how you want it to be.”	
	Location-based notifications	“You’ll get a notification like oh there’s a concert happening in the park. Or like oh this museum is not charging today. Something like that. It all depends on where you’re at. So it’s around your surroundings.”	

## Discussion

### Principal Findings

We queried adolescents who use cannabis about specific aspects of a mobile technology–based intervention, including content, duration, user interface, accessibility, support (peer and clinician), and current/past use of health behavior change apps, to inform development of a cannabis cessation intervention that would increase treatment engagement by youth in substance use treatment.

Thetwomost common themes in intervention development that were expressed by participants were (1) monitoring reduction of cannabis use over time and (2) providing rewards for successful reduction and cessation. Adolescents indicated that integration of CM would be appealing. Previous research suggests that CM can improve effectiveness of substance use treatment for youth and increase engagement in treatment [33-36], including for youth with CUDs [37]. Surprisingly, participants in our study indicated that nonmonetary rewards would be the most rewarding; this included receiving emojis, similar to *streaks* in Snapchat, which many expressed would be motivating enough to maintain abstinence. Participants also indicated that being rewarded with *Groupons* for prosocial activities, such as movie theater or concert tickets, where one would be unable to use substances, would be appealing. Participants also expressed preference for receiving information and/or tickets for activities and events specific to their region, as well as rides to and from these activities and indicated that they would approve of integrating geolocation tracking services into the intervention to individualize rewards. Ironically, this is despite research showing that 46% of adolescents are likely to turn off or disable geolocation features associated with downloaded apps because of concerns of privacy [15]. The difference between our findings and those previously reported may be because of expectations of privacy starting at the point of download. For our purposes, there is an expectation that personal information would be collected to treat a disorder, whereas adolescents in the Pew Research study indicated downloading free apps for entertainment purposes where there is no expectation that personal information is needed to reach the desired outcome. Furthermore, adolescents may be more willing to share personal information, including location, when it results in a reward. Other studies have found that privacy and confidentiality were of less concern to high-risk adolescents if addressed at the beginning of a mHealth intervention [38]. Behavioral interventions for adolescent substance use treatment incorporate encouragement of prosocial activities to avoid substance use, with increased efficacy of intervention, if adolescents are able to increase engagement in these activities. The adolescents enrolled in this study also independently identified engagement in alternative, prosocial activities as a way to decrease use, and indicated updates to traditional thoughts about types of activities (eg, school-based sports or clubs) to technology-accessed activities specific to their lives and communities.

Privacy was an interesting theme that emerged in an isolated context, but also within discussion of other content areas. When asked directly about privacy and others’ potential to have access to participants’ data, knowledge of their involvement in the intervention, or location-based information available to researchers/clinicians for intervention delivery, participants almost uniformly had negative views. However, with regard to rewards and peer resources, participants voluntarily and spontaneously proposed offering personal information and inclusion of location-based services to gain psychosocial and other intangible rewards, as well as tangible rewards.

The theme of individualizing the app was also discussed in other domains. Individualization of mHealth interventions has been identified in previous studies as important to high-risk adolescents seeking treatment [38]. In our study, adolescents indicated their desire to individualize all aspects of the app, including the user interface, frequency, timing, tone and content of notifications and messages, and the level of clinician, peer, or text-based responsiveness to fluctuations in mood states and substance use. Dynamic interventions that address these factors are currently lacking from mHealth interventions, especially those tailored to youth. The majority of mHealth interventions for substance using and/or at-risk youth are static, text message–based interventions that do not address differences in baseline severity of substance use, psychiatric symptoms, and/or adapt to changes in these behaviors or symptoms over time.

Themes identified by adolescents who use cannabis, the target population for this app-based cannabis use intervention, are consistent with research suggesting that intervention efficacy may be increased by developing selective, highly specific interventions incorporating content that addresses risk factors in at-risk youth and are presented on interactive platforms [39].

### Limitations

There are several limitations to this study. As we sampled adolescents who use cannabis from one city in Southern California, it was not representative of the broader adolescent population. In addition, we had a low number of racial and ethnic minorities in our sample, further limiting generalizability. With regard to uses of mobile technology, we did not query adolescents about alternate platforms for intervention other than an app delivered via smartphone. Although the literature shows that adolescents’ primary mode of mobile technology use are smartphones, we did not query participants about use or acceptability of other types of technologies. Finally, we did not query youth on general methods of access to the app, as the goal is to integrate this into extant treatment paradigms for users engaged in substance use treatment. Despite these limitations, the qualitative approach allowed us to further probe the complexities and context of components of intervention development to understand adolescent technology use, and motivations for appeal of component content, more in-depth.

### Future Directions

This app, incorporating mobile CBT, is being developed as an adjunct to TAU for adolescent cannabis users aged between 13 and 18 years who have 9 to 12 weeks of substance use treatment remaining at the time of study entry (to ensure we enroll youth before TAU impacts substance use). Participants will be recruited from local adolescent substance use treatment clinics. The youth clinics provide case management and individual and group counseling intwotracks, outpatient drug-free groups (meet daily, Monday-Friday for 1.5 hours after school) and day care habilitative groups (intensive daily, Monday-Friday, half-day program), with adjunctive family therapy, parent and youth support meetings, and aftercare counseling, as needed. Random urine toxicology is done to confirm abstinence. Youth are enrolled in treatment for 3 months, with 1 month added to treatment length for each positive urine toxicology screen. An active cohort (TAU+biosensor) will be matched by primary substance of use to a cohort receiving TAU alone (matched comparison cohort data collected through electronic medical record [EMR]/chart review) in an external control design. We will assess and compare groups on the primary outcome of point prevalence abstinence (total number throughout treatment and number of consecutive weeks) for primary substance of use (substance for which the youth meets criteria for SUD), and secondary outcome of point prevalence abstinence for total number of substances used. These outcomes will be collected for the TAU group by reviewing their medical records. Collecting primary outcome comparison data through EMR/chart review will provide us with an important opportunity to identify systems-related considerations of cross-database information sharing, privacy, confidentiality, and other issues that must be resolved before launching a larger-scale study, in which obtaining collateral EMR/chart data will be essential to supporting treatment outcomes research trials.

### Conclusions

Adolescent cannabis use initiation is linked to negative long-term health effects [38,39], with likely greater impact because of rising delta-9-tetrahydrocannabinol concentrations [40,41]. Technology-based substance use interventions appear promising in adult and college-aged populations, but few are adapted for youth, and none specifically targeted for adolescents with CUD. Behavioral interventions for adolescent cannabis use have demonstrated limited success, with high rates of relapse and loss of gains at follow-up [11,13]. mHealth interventions allow for examination of behaviors, ecological and mental states, environments, and social networks contributing to, and resulting from, adolescent substance use in real time, with near instantaneous response and intervention. Collectively, real-time data can shape targeted treatment around individual risk factors for problematic use. Furthermore, mHealth interventions have the potential to address current barriers to treatment, including cost, stigma, and access to providers with adequate training in utilizing/implementing behavioral interventions specifically targeting substance use, as well as increasing overall reach and scalability of interventions.

We have presented novel end user–informed data about the content, format, structure, privacy, and accessibility of an app-based substance use treatment for adolescents that may inform more successful interventions among this high-risk population. In this study, the sample of adolescents who use cannabis indicated a desire for an individualized app, with highly visual components consistent with apps that they already use and escalating rewards associated with individual progress. Furthermore, although response to use of geolocation services in the context of discussion of privacy was mixed, adolescents endorsed approval of sharing GPS information in the context of discussion pertaining to individualized rewards and connecting with peers. Identifying and developing intervention content on platforms highly utilized by target population, incorporating skill development (eg, saying no, coping with negative feeling, engagement in prosocial activities) via novel technological means, and integrating salient environmental rewards may increase intervention efficacy, and thus may improve substance use treatment outcomes. Further study of ethical and privacy implications of novel technological approaches are needed in this population. However, methods of ensuring that data are transmitted and stored in a manner that complies with Health Insurance Portability and Accountability (HIPAA) requirements are possible by ensuring that (1) all information from mobile to server will be via https/SSL (secure sockets layer), (2) stored data are encrypted and deleted when no longer required, (3) servers on the cloud are HIPAA compliant and regularly patched with security updates, stored in a secure facility, and (4) provision of a clear privacy policy and (5) strong passwords with expiration periods are mandated.

## Abbreviations

CBT: cognitive behavioral therapy
CM: contingency management
CUD: cannabis use disorder
EMR: electronic medical record
GPS: global positioning system
HIPAA: Health Insurance Portability and Accountability
MET: motivational enhancement therapy
mHealth: mobile health
SDUSD: San Diego Unified School District
SMS: short message service
TAU: treatment-as-usual
